# Coronary computed tomographic angiography and atherosclerosis: prognostic validation of coronary scores in a Slovenian cohort

**DOI:** 10.2478/raon-2026-0004

**Published:** 2026-01-13

**Authors:** Tadeja Poropat Flerin, Borut Jug, Daniel Kosuta

**Affiliations:** Institute of Radiology, University Medical Center Ljubljana, Ljubljana, Slovenia; Department of Vascular Diseases, University Medical Center Ljubljana, Ljubljana, Slovenia

**Keywords:** coronary computed tomographic angiography, atherosclerosis, coronary scores

## Abstract

**Background:**

Coronary computed tomographic angiography (CCTA) provides information on coronary atherosclerosis burden and extent. In the present analysis, we compared the prognostic impact of coronary scores (maximal coronary stenosis, segment involvement score [SIS] and segment stenosis scores [SSS], and the CCTA-modified Duke score).

**Patients and methods:**

We retrospectively reviewed CCTA images of patients with suspected coronary obstruction and excluded patients who underwent planned revascularization. Using Cox multivariate analysis, we estimated the hazard ratio (HR) with 95% confidence intervals (CI) for different coronary scores to predict death, myocardial infarction, and late unplanned revascularizations (as individual and composite endpoints). Model performance was evaluated using area under time-dependent receiver operating characteristic curves (AUC).

**Results:**

We included 750 patients (median age 61 years, 54% women) with a median follow up 1,465 days. Unadjusted HR for major cardiovascular events ranged from 3.87 (95% CI 1.49–10.0, p = 0.005) for obstructive disease (> 50% stenosis in any vessel) to 1.17 (1.09–1.25, p < 0.001) for SIS (each additional segment involved). Predictions remained significant for all endpoints and after adjusting for coronary artery calcium score and risk factors. Area under curve (AUC) for coronary stenosis was 0.77 (95% CI 0.71–0.82), for SIS was 0.77 (95% CI 0.72–0.83), for SSS was 0.77 (95% CI 0.71–0.82), and for Duke score was 0.67 (95% CI 0.61–0.74).

**Conclusions:**

Our study has confirmed that coronary atherosclerosis burden and extent independently predict major cardiovascular events in patients who had undergone CCTA, but were not referred for invasive diagnostic procedures and revascularization.

## Introduction

Cardiovascular diseases (CVD) remain the leading cause of mortality globally.^1^ Despite a dramatic decline in premature and age-standardized CVD mortality over the past three decades, ageing populations and increasing prevalence of cardiometabolic risk factors continue to fuel the persisting epidemiological burden of CVD.^2^ Atherosclerosis remains the predominant driver of CVD, with the majority of CVD deaths resulting from ischemic heart disease and stroke.^1,2^ Identification of individuals at high risk of CVD events, such as myocardial infarction and stroke, thus represents a pivotal step in addressing the burden of CVD. In practice, cardiovascular risk assessment for CVD is based on conventional risk factors and risk factor-derived estimations, such as the Framingham score or the SCORE2/SCORE2-OP. While risk factor-derived estimation is indispensable for CVD risk prediction and management, imaging methods for direct visualization of atherosclerotic vascular disease provide an increasingly available approach for improved CVD risk stratification.

At present, cardiac computed tomography (CT) is the most accurate non-invasive modality for direct visualization of coronary atherosclerosis. Non-contrast cardiac CT for detection of coronary calcifications (i.e., calcium scoring, CACS) and contrast-enhanced cardiac CT for detection of coronary plaques (coronary computed tomographic angiography, CCTA) are the most widely used cardiac CT modalities. CACS provides a guideline-endorsed tool for improved risk stratification in asymptomatic individuals at intermediate CVD risk - i.e., detection of subclinical atherosclerosis.^3,4^ Conversely, CCTA in mainly used in symptomatic individuals - i.e., patients with chest pain or other symptoms suggestive of coronary obstruction undergo CCTA imaging to appreciate whether invasive angiography and revascularization is indicated (‘gate-keeping’).^5,6^ However, CCTA also provides additional appreciation of non-obstructive coronary plaques.^7^ Given the expanding role of CCTA, the finding of non-obstructive coronary artery disease (CAD) has several implications, both in terms of CVD risk assessment and in terms of preventive management.

Coronary plaque burden is the most important marker of increased CVD risk and can be indicated by degree of stenosis (e.g., a 50% stenosis indicates a heavier atherosclerotic burden than a 30% stenosis), anatomic extent (e.g., involvement of more coronary arteries/segments indicates a heavier atherosclerotic burden than single segment coronary plaque), and anatomic location (e.g., disease of the left main coronary artery or proximal segments indicates higher risk then involvement of distal segments).^8^ Coronary plaque burden can be captured by several scores, such as the coronary artery disease-reporting and data system (CAD-RADS) – grading stenosis from 0/none to 5/total obstruction), segment involvement score (SIS)–summarizing the number of affected coronary segments up to a total of 15) and segment stenosis score (SSS)–multiplicating the grade of stenosis with the number of affected segments), or the modified Duke coronary artery disease score (accounting for grade of stenosis, number of affected vessels, and location). We have previously reported on the prognostic significance of obstructive vs. non-obstructive CCTA-detected coronary artery disease.^9^ in the present analysis, we sought to validate multiple CCTA-derived coronary scoring systems within a real-world cohort of patients undergoing CCTA. This is particularly important given the growing clinical use of CCTA and the need for accurate risk stratification in patients who are not referred for invasive management but still require intensive preventive care. Unlike prior studies, our analysis focuses exclusively on a non-invasive management population, providing novel insights into the long-term prognostic performance of CCTA-derived scores in a conservatively managed, real-world setting.

## Patients and methods

### Study design, population, and outcomes

This was a longitudinal study with retrospective design focusing a time-to-event analysis of consecutive patients undergoing CCTA at the University Medical Centre of Ljubljana between 2010 and 2016. We excluded patients with non-diagnostic CCTA because of elevated calcium score or artefacts in 2 or more coronary segments, patients with known coronary artery disease (after coronary artery bypass grafting or percutaneous revascularization), patients undergoing CCTA for aortic stenosis (i.e., transcatheter aortic valve implantation protocol) or adult congenital heart disease, and patients referred for downstream invasive coronary management triggered by CCTA. Researchers blinded to the CCTA results conducted follow-up for mortality (through national vital status database) and for nonfatal events (direct/telephone follow up with patients/next of keen/general practitioner, or review of medical records). The study was approved by the National Medical Ethics Committee, Republic of Slovenia (approval number 62/06/14).

### Computed tomographic coronary angiography

Coronary computed tomographic angiography scanning was performed using a 128-row multiple detector computed tomography scanner (Siemens Biograph mCT, Siemens Medical Solutions Inc). For patients who were ≥ 50 years of age, calcium scoring was performed upfront, and if it was reasonably low (Agatston score < 300), angiographic protocol was continued. Cardiac CT was performed using a 128-row multiple detector computed tomography scanner (Siemens Biograph mCT, Siemens Medical Solutions Inc). CACS was performed upfront followed by electrocardiogram gated, prospective scanning protocol with intravenous administration of contrast and following premedication with sublingual glyceryl trinitrate and metoprolol at the discretion of physician performing the CCTA. Data sets were reconstructed and 3-dimensional images analysed using dedicated software. Coronary atherosclerosis was defined as any visualized lesion within, or adjacent to, the arterial lumen that could be differentiated from surrounding anatomical structures.

### Coronary artery disease grading and scoring

Atherosclerotic lesions were graded using the following methods/scores: CAD-RADS stenosis grading, obstruction level categorization, SIS, SSS, and modified Duke coronary artery disease index (Table 1).

**TABELA 1.** Coronary risk score overview

Scoring system	Definition	Scoring range	Used in model(s)
**Maximal coronary stenosis**	Maximum stenosis severity observed across LAD, LCX, RCA according to CAD-RADS definition	0 - No stenosis 1- 1–24% stenosis 2 - 25–49% stenosis 3 - 50–69% stenosis 4 - 70–99% stenosis (≥ 50% if LM involved) 5 - Occlusion	e.g., 30–49% stenosis of LAD (score = 2) and 70–99% stenosis of proximal RCA (score = 4) yields a maximal coronary stenosis of 4
**Obstruction category**	Categorized as none, non-obstructive, or obstructive	None - No stenosis non-obstructive - 1–69% stenosis Obstructive - ≥ 70 stenosis (≥ 50% if LM involved)	e.g., 30–49% stenosis of LAD (score = 2) and 70–99% stenosis of proximal RCA (score = 4) yields a categorisation of obstructive CAD
**Segment involvement score (SIS)**	Number of coronary segments with any stenosis; a segment is counted if there is any measurable stenosis (typically corresponds to ≥1 on a 0–5 scale)	0–15	e.g., isolated involvement of mid LAD and proximal RCA yields a SIS score of 2
**Segment stenosis score (SSS)**	Sum of all stenosis severity scores across 15 coronary segments	0–75	e.g., 30–49% stenosis of the mid LAD (score = 2) and 70–99% stenosis of the proximal RCA (score = 4) yields a SIS of 6
**Duke CAD Score**	Number of major vessels with ≥ 70% stenosis (score ≥ 4), + 1 if LADp also ≥ 70%	0 - None or single < 5 0% stenosis 1 - ≥ 2 stenoses 30% to 49% (one proximal) 2 - 2 stenoses 50–69% or 1 stenosis ≥ 70% 3 - 3 stenoses 50-69% or 2 vessel or proximal LAD stenosis ≥70% 4 - 3 stenosis ≥ 70% or 2 vessels with proximal LAD stenosis ≥ 70% 5 - ≥ 50% LM stenosis	e.g., 30–49% stenosis of LAD (score = 2) and 50–69% stenosis of proximal RCA (score = 4) yields a Duke CAD score of 2

1 CAD = coronary artery disease; CAD-RADS = coronary artery disease-reporting and data system; LAD = *left anterior descending coronary artery; LADp = left anterior descending coronary artery* blocked; *LCX = left circumflex coronary artery; LM* = left main coronary artery; *RCA* = right coronary artery; SIS = segment involvement score

### Outcomes

Outcomes of interest included i) death, ii) a composite of death and non-fatal myocardial infarction (i.e., two-point major cardiovascular event, 2-point MACE), and a iii) composite of death, non-fatal myocardiac infarction, or hospitalization for unstable angina with or without unplanned revascularization (i.e., 3-point MACE).

### Statistical analyses

Summary descriptive statistics were expressed as medians (and interquartile ranges) for continuous variables and as counts (and percentages) for categorical variables. Exploratory comparisons between independent groups (e.g., baseline characteristics) were conducted using the Kruskal-Wallis test (for continuous variables) or the χ^2^ test (for categorical variables). Missing data in continuous and categorical variables were treated as missing-atrandom and imputed with predictive mean matching (PMM), incorporating selected predictors (age, sex, and cardiovascular risk factors).

Survival analysis was performed with Kaplan-Meier curves and Cox proportional hazards models. Cox models were fitted to estimate the association of each score (maximal coronary stenosis, SIS, SSS, and Duke score) with and time to each outcome (death, 2-point MACE, 3-point MACE). Cox models were adjusted sequentially: unadjusted models (score only), adjusted for CACS, fully adjusted model (for CACS, age, sex, arterial hypertension, diabetes mellitus, and smoking). Model performance was evaluated using Harrell’s concordance index (C-index) and time-dependent ROC curves at 3 and 5 years. Differences between AUCs were compared using z-tests with standard error propagation. Long-term risk (10-year) of MACE across obstruction categories was estimated using a log-normal survival model. Predictions were plotted with 95% confidence intervals. Statistical significance was set at twotailed *p* < 0.05. Statistical analyses were performed with R version 4.4.1 (R Foundation for Statistical Computing, Austria).

## Results

**FIGURE 1.**
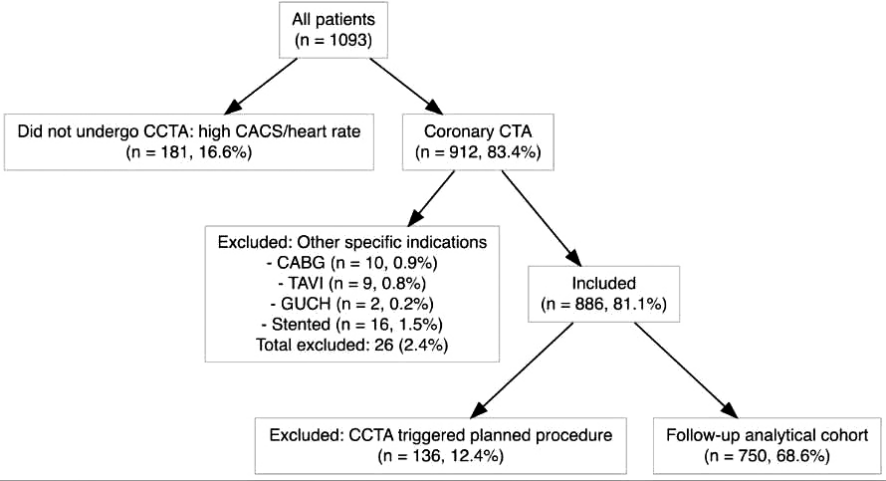
Flowchart of patient enrollment process. CABG = coronary artery bypass graft; CACS = calcium scoring; CCTA = computed tomographic angiography; CTA = computed tomographic angiography; GUCH = grown-up congenital heart disease; TAVI = transcatheter aortic-valve implantation

The analytical cohort comprised 750 individuals (median age 61 years, 54% women (Table 2 and Figure 1). During a median follow-up time of 1,465 days (interquartile range 1,158–2,032 days), 26 (3.5%) patients died, 27 (3.6%) experienced a 2-point composite event (death or myocardial infarction) and 35 (4.7%) experienced a 3-point composite event (death, myocardial infarction, or unplanned revascularization, yielding an overall event rates of 0.86%, 0.9% and 1.5% per 100 patient-years, respectively.

**TABELA 2.** Baseline characteristics

Characteristic		N = 750^1^
Sex (male)		346 (46%)
Age (years)		61 (52, 69)
Arterial hypertension		70 (9.3%)
Diabetes mellitus		25 (3.3%)
Dyslipidemia		43 (5.7%)
Smoking		28 (3.7%)
CACS	*Zero*	531 (71%)
	*1–99*	123 (16%)
	*100–299*	51 (6.8%)
	*≥ 300*	45 (6.0%)
Stenosis	*None*	542 (72%)
	*1–24% stenosis*	35 (4.7%)
	*25–49% stenosis*	61 (8.1%)
	*50–69% stenosis*	40 (5.3%)
	*70–99% stenosis*	59 (7.9%)
	*Occlusion*	13 (1.7%)
Obstruction	*None*	542 (72%)
	*Non-obstructive*	136 (18%)
	*Obstructive*	72 (9.6%)
^1^n (%); Median (IQR); Mean (SD)		

1 CACS = calcium scoring; IQR = interquartile range; SD = standard deviation

**FIGURE 2.**
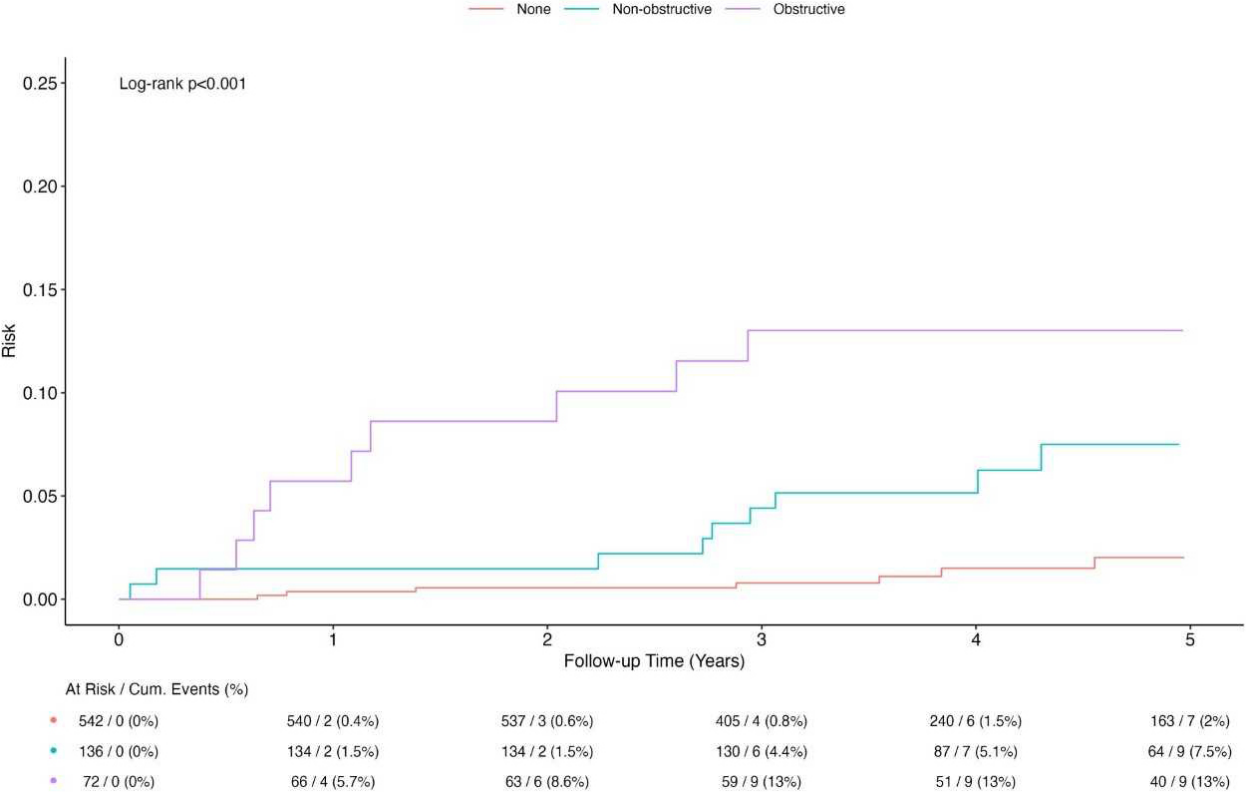
Five-year survival curves for no atherosclerosis, non-obstructive and obstructive atherosclerosis (Log-scale).

Presence of coronary artery disease expectedly predicted time to death, non-fatal myocardial infarction and hospital admission for unstable angina; severity of stenosis and extent of coronary artery disease was associated with increasing risk, as depicted by increasing risk in no atherosclerosis, non-obstructive atherosclerosis, and obstructive atherosclerosis (Figure 2). Estimated long-term risk (10-year) of MACE was 3% (95% CI 0–14%), 11% (95% CI 3–27%), and 21% (95% CI 9–39%) for none, non-obstructive and obstructive coronary artery disease, respectively.

All coronary scores predicted death, 2-point MACE, and 3-point MACE (Table 3). Robust categorizations yielded largest estimates (e.g., 3.86-fold increase in the risk of death with obstructive vs. no coronary atherosclerosis), whereas ordinal scores provided more granular risk appreciation (e.g., a 16% increase in the risk of death for every additional segment with stenosis). The risk estimation for all scores diminished, but remained significant after multivariate adjustment (e.g., from 16% unadjusted to 11% fully adjusted for every additional segment with stenosis).

**TABELA 3.** Cox models for different coronary computed tomographic angiography (CCTA)-derived scoring systems and their association with clinical endpoints

	CV Death				MACE			MACE plus unplanned revascularization		
	Model	HR	CI	p	HR	CI	p	HR	CI	p
**Segment involvement score (SIS)**	Base model	1.75	1.33–2.29	< 0.001	1.80	1.39–2.34	< 0.001	1.84	1.47–2.31	< 0.001
	Plus calcium score (log)	1.72	1.13–2.61	0.011	1.72	1.15–2.59	0.008	1.56	1.09–2.24	0.015
	Full model	1.56	1.03–2.37	0.038	1.61	1.07–2.42	0.023	1.69	1.17–2.45	0.005
**Segment stenosis score (SSS)**	Base model	1.16	1.08–1.24	< 0.001	1.17	1.09–1.25	< 0.001	1.18	1.11–1.25	< 0.001
	Plus calcium score (log)	1.13	1.01–1.26	0.026	1.14	1.03–1.26	0.010	1.12	1.03–1.23	0.009
	Full model	1.11	1–1.24	0.057	1.13	1.02–1.25	0.024	1.13	1.03–1.24	0.009
**Modified Duke CAD score**	Base model	1.48	1.14–1.92	0.003	1.54	1.2–1.96	< 0.001	1.66	1.35–2.02	< 0.001
	Plus calcium score (log)	1.25	0.9–1.73	0.192	1.27	0.93–1.75	0.132	1.34	1.03–1.74	0.032
	Full model	1.18	0.85–1.64	0.324	1.21	0.88–1.67	0.237	1.35	1.01–1.81	0.041
**Maximal coronary stenosis grade**	Base model	1.58	1.28–1.95	< 0.001	1.63	1.33–2	< 0.001	1.70	1.42–2.04	< 0.001
	Plus calcium score (log)	1.64	1.22–2.19	< 0.001	1.68	1.26–2.23	< 0.001	1.66	1.28–2.15	< 0.001
	Full model	1.54	1.16–2.04	0.003	1.58	1.2–2.09	0.001	1.66	1.3–2.12	< 0.001
**Presence of any non-obstructive plaque**	Base model	3.86	1.49–10.03	0.006	3.87	1.49–10.06	0.005	2.78	1.15–6.73	0.023
	Plus calcium score (log)	4.44	1.51–13.05	0.007	4.23	1.44–12.48	0.009	2.43	0.87–6.82	0.092
	Full model	3.53	1.19–10.44	0.023	3.57	1.21–10.6	0.022	3.24	1.16–9.08	0.025

1 CAD = coronary artery disease; CI = confidence interval; CV = cardiovascular; FR = hazard ratio; MACE = major cardiovascular event; SIS = segment involvement score; SSS = segment stenosis scores

**FIGURE 3.**
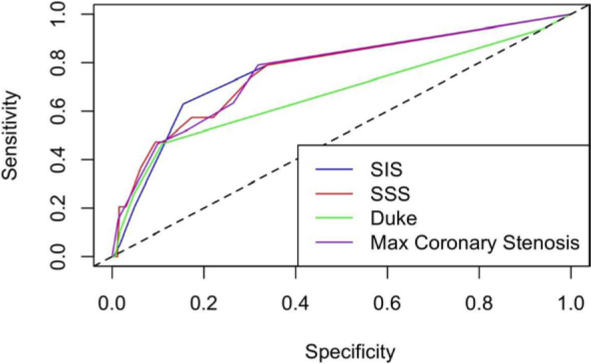
Predictive accuracy of coronary scores: area under curve (AUC) comparison for stenosis, segment involvement score (SIS), segment stenosis scores (SSS) and Duke score.

For coronary analysis yielding ordinal scores, AUC for coronary stenosis was 0.77 (95% CI 0.71–0.82), for SIS was 0.77 (95% CI 0.72–0.83), for SSS was 0.77 (95% CI 0.71–0.82) and for Duke score was 0.67 (95% CI 0.61–0.74) (Figure 3).

Although SIS, SSS, and stenosis severity scores demonstrated comparable discriminative performance (AUC 0,77), their relative simplicity and reproducibility may guide their preferred integration into routine clinical workflows. The lower predictive accuracy of the Duke score highlights the added value of more detailed, segment-based assessments of coronary plaque burden.

Predictive accuracy for coronary stenosis, SIS, and SSS was retained throughout 3 years of follow-up, while the predictive accuracy for Duke score diminished after 1 year of follow-up.

## Discussion

Our analysis explored the prognostic role of different CCTA coronary scores in an unselected population of patients with suspected coronary artery disease. All scores reliably predicted cardiovascular prognosis, although maximal stenosis and summary segment involvement (the SIS and SSS score) outperformed the modified Duke CAD score. Overall, the presence and extent of coronary atherosclerosis, as appreciated by CCTA, represented the single most important marker of cardiovascular risk; in our patient population, which excluded individuals referred for downstream invasive angiography (i.e., were filtered out by the gate-keeping function of CCTA), the presence and extent of coronary atherosclerosis warrants aggressive risk management, including intensive preventive therapy.

Presence of atherosclerosis was associated with a significant risk. The projected 10-year risk for a major cardiovascular event ranged from 11% for non-obstructive to 21% for obstructive atherosclerosis, well above the 7.5% threshold currently proposed for high CVD categorization. Our results reflect previous studies, which have associated the degree of obstruction with incremental cardiovascular risk.^9–12^ Of note, the observed risk differences between obstructive and non-obstructive CAD in CCTA studies were strongly impacted by considering revascularizations as endpoints (as CCTA-detected obstruction triggers downstream revascularization, the difference was likely due to referral bias).^8,13^ In our analysis, downstream invasive angiography and management were excluded; nonetheless, presence as well as extent of atherosclerotic coronary involvement was associated with the future risk of hard cardiovascularendpoints (cardiovascular death and myocardial infarction) as well as unplanned revascularization (for unstable or progressive anginal symptoms).

Plaque burden and anatomic extent are additional determinants of risk. In our analysis, both the number of coronary segments with plaque (SIS) and stenosis (SSS) reliably were associated with cardiovascular death and composite endpoints. While risk estimations decreased after multivariate adjustment (e.g., the HR for each additional segment decreased from 1.8 to 1.6 after adjusting for calcium score and risk factors), the persisting prognostic power of coronary artery disease imaging warrants intensive management. Our results align with previous reports.^14,15^ Based on 1,127 patients undergoing CCTA for symptoms of CAD, SIS is associated with a 1.23-fold increase (95% CI 1.13–1.34) and SSS is associated with a 2-fold increase (95% CI 1.48–2.67) in all-cause mortality.^14^ In fact, detecting diffuse non-obstructive atherosclerosis dominates over detecting focal significant stenosis in the prediction of major cardiovascular events.^16,17^ Plaque burden and degree of stenosis are routinely reported when documenting CCTA-detected atherosclerosis, in line with the CAD-RADS 2.0 reporting system.^18^

Current guidelines recommend CCTA for patients with symptoms suggestive of coronary obstruction, primarily for “gate-keeping” for invasive coronary angiography.^19,20^ However, CCTA diagnostic findings (e.g., obstructive or non-obstructive CAD) reliably predict the rates of future cardiovascular events.^8,13,21^ At present, CCTA is also the only evidence-based non-invasive test for coronary artery disease. The SCOT-HEART trial randomized symptomatic patients to either CCTA or standard clinical care and showed that a CCTA-based strategy reduced the risk of coronary death or nonfatal myocardial infarction^22^, which was sustained long after the trial was concluded, primarily through intensive preventive management.^23^ The degree of obstruction is but one of the markers of atherosclerotic burden; in fact, the majority of acute coronary events may derive from extensive non-obstructive plaques.^17^ Thus, a finding of coronary atherosclerosis – even in the absence of indications for invasive diagnostics or interventions – should prompt the initiation and sustained implementation of intensive cardiovascular risk reduction strategies.

Given the association between non-obstructive plaque burden and an increased 10-year risk of major adverse cardiovascular events (MACE), clinicians should consider the initiating or intensifying preventive measures-including statin therapy, aspirin when appropriate and lifestyle modification-in patients with intermediate clinical risk who demonstrate diffuse atherosclerosis on CCTA, regardless of the degree of stenosis.

Limitations our study primarily pertain to its retrospective observational design at a single tertiary center. Firstly, observational design provides inference on association, not causation. Importantly, while the prognostic impact of subsequent revascularization can be addressed by excluding patients who were referred for invasive procedures and a separate analysis for unplanned revascularization was conducted, the impact of preventive medication was not available for the present analysis. Secondly, the retrospective acquisition of research data introduced missing values, which were addressed using imputation methods. Thirdly, while inclusion of consecutive patients provides a fair degree of external validity, results from patients referred to a tertiary center may not be entirely generalizable to other patient populations. Furthermore, patients with obstructive coronary artery disease included in our study who were not referred for invasive diagnostic evaluation, often demonstrated clinical stability without signs of ischemia, had comorbidities or personal preferences that contraindicated invasive management, or had functional assessment results indicating limited potential benefit from revascularization. While this reflects real-world clinical decision-making, it may introduce selection bias that could affect the observed risk estimates and limit the generalizability of the findings to the broader population of individuals with obstructive coronary disease.

## Conclusions

Overall, our study has confirmed that coronary atherosclerosis burden and extent provide a strong predictor of major cardiovascular events in patients who had undergone CCTA but were not referred for invasive diagnostic procedures and revascularization. The rate of major cardiovascular events suggests that this patient population is at high cardiovascular risk and that a CCTA finding should be regarded as an important prognostic marker, even after adjusting for age, sex, calcium score, and conventional risk factors.
